# Correction: The “LLM World of Words” English free association norms generated by large language models

**DOI:** 10.1038/s41597-025-05265-5

**Published:** 2025-06-05

**Authors:** Katherine Abramski, Riccardo Improta, Giulio Rossetti, Massimo Stella

**Affiliations:** 1 University of Pisa, Department of Computer Science, Pisa, Italy; 2 University of Trento, Department of Psychology and Cognitive Science, Trento, Italy; 3 National Research Council of Italy, Institute of Information Science and Technologies, Pisa, Italy

**Keywords:** Computer science, Psychology

Correction to: *Scientific Data* 10.1038/s41597-025-05156-9, published online 16 May 2025

In this article Massimo Stella (massimo.stella-1@unitn.it) was omitted as a corresponding author. Second, The Acknowledgements section was missing from this article and should have read 'This work is supported by: (i) the Italian Ministry of University and Research (MUR) through the Department of Excellence grant awarded to the Department of Psychology and Cognitive Science, UniTrento; (ii) the COGNOSCO research grant funded by UniTrento (PS_22_27); (iii) the CALCOLO research grant funded by Fondazione VRT; (iv) SoBigData.it which receives funding from the European Union – NextGenerationEU – National Recovery and Resilience Plan (Piano Nazionale di Ripresa e Resilienza, PNRR) – Project: “SoBigData.it – Strengthening the Italian RI for Social Mining and Big Data Analytics” – Prot. IR0000013 – Avviso n. 3264 del 28/12/2021; (v) EU NextGenerationEU programme under the funding schemes PNRR-PE-AI FAIR (Future Artificial Intelligence Research)’. The original article has been corrected.

